# Genetic Features of Plasmid- and Chromosome-Mediated *mcr-1* in *Escherichia coli* Isolates From Animal Organs With Lesions

**DOI:** 10.3389/fmicb.2021.707332

**Published:** 2021-08-05

**Authors:** Zengyuan Liu, Yingqiu Liu, Wei Xi, Shuangshi Liu, Jia Liu, Hailong Mu, Beibei Chen, Hao He, Yunpeng Fan, Wuren Ma, Weimin Zhang, Mingzhe Fu, Juan Wang, Xiaoping Song

**Affiliations:** ^1^College of Veterinary Medicine, Northwest A&F University, Yangling, China; ^2^Qingdao Adverse Drug Reaction Monitoring Center, China Qingdao Institute for Food and Drug Control, Qingdao, China

**Keywords:** *Escherichia coli*, *mcr-1*, sequence type, T4SS, integrons and transposons

## Abstract

The genomic context of the *mcr-1* gene in *Escherichia coli* from animal feces has been widely reported. However, less is known about the *mcr-1*-carrying plasmid characteristics and other functional regions of *Escherichia coli* isolates from animal organs with lesions. The present study investigated the antimicrobial resistance, population structure, and genetic features of *mcr-1*-positive *Escherichia coli* strains isolated from animal organs with lesions. The antimicrobial susceptibility testing indicated that 24 *mcr-1*-positive *Escherichia coli* isolates were resistant to at least three or all antimicrobial categories. MLST analysis suggested that the dominant clone complexes (CC) were mainly CC156, CC448, and CC10. In addition, ST10596, a newly discovered sequence type in swine, failed to be classified. Meanwhile, the *mcr-1* gene located on the different plasmids was successfully transferred to the recipients, and whole-genome sequencing indicated the *mcr-1* gene was embedded in *mcr-1-pap2* cassette but not flanked by IS*Apl1*. The *mcr-1* gene is located on the chromosome and embedded in Tn*6330*. Furthermore, *NDM-5* was found on the IncX3-type plasmid of J-8. The *virB6* and *traI* gene of type IV secretion system (T4SS) were truncated by IS*2* and IS*100* and located on the IncX4- and the IncHI2/HI2A/N-type plasmids, respectively. The multidrug-resistant (MDR) region of IncHI2/HI2A/N-type plasmids contained two class 1 integrons (In*0*, In*640*) and four composite transposons (Tn*4352*, Tn*6010*, cn_4692_IS*26*, cn_6354_IS*26*). Overall, 24 *mcr-1*-positive *Escherichia coli* isolates in our study showed MDR, or even extensively drug resistant (XDR), and exhibited population diversity. The T4SS gene truncation by the insertion sequence may affect the efficiency of plasmid conjugative transfer. Furthermore, the class 1 integrons and composite transposons in the MDR region of IncHI2/HI2A/n-type plasmid contributed to the multireplicon plasmid formation, the acquisition, and transfer of antimicrobial resistance genes (ARGs).

## Introduction

The overuse and misuse of antimicrobials have led to the rapid development of resistance in Gram-negative bacteria, and this has now become a serious public health crisis (Martin et al., 2020). Among these bacteria, *Escherichia coli* is the most common pathogen which causes animal and human diseases on a global scale while being a major cause of multiple acquired infections (Aslam et al., 2018). Over the past few years, the emergence and common occurrence of extended-spectrum beta-lactamase (ESBL) – and carbapenemase-producing *E. coli* have result in reduced effectiveness of antimicrobial treatment (Hordijk et al., 2020), such that doctors are now considering alternative antimicrobials, such as colistin.

For many years, colistin (polymyxin E) has been used in animal production for the treatment and prevention of infectious diseases. It was subsequently identified as a form of last-resort treatment against severe human infections caused by multidrug- and carbapenem-resistant Gram-negative bacteria (Kempf et al., 2016; Poirel et al., 2017). Colistin resistance was previously thought to be chromosome mediated, which mainly related to the point mutations of two-component systems (TCSs) PhoP/PhoQ and PmrA/PmrB and can transmit colistin resistance through vertical transmission (Olaitan et al., 2014). It was only in 2015 that the plasmid-mediated *mcr-1* gene was first described in *E. coli* and *Klebsiella pneumoniae*. Both species were, respectively, isolated from pigs and humans in China and showed the horizontal transfer of colistin resistance (Liu et al., 2016). The emergence of this gene has further reduced the effectiveness of the alternative antimicrobials, thereby posing new challenges to public health. Since then, colistin-resistant *mcr-1*-positive *E. coli* isolates have been reported in the environment, humans, animals, and food worldwide (Falgenhauer et al., 2016; Luo et al., 2017; Shen et al., 2018). Numerous researches have shown that the high clonal diversity was observed among *mcr-1*-positive *E. coli* (MPECs) and mainly belonged to the common dominant clone complexes, such as ST10, ST48, ST165, etc. (Matamoros, 2017; Bai et al., 2018). Among these, ST10 is the most common transmission clone group and frequently disseminates through different means (Sellera et al., 2017; Patiño-Navarrete et al., 2020).

The *mcr-1* gene is found in multiple plasmid types, including IncI2, IncX4, IncX3-X4, IncHI1, IncHI2, IncP, IncY, IncK2, IncFI, IncFI, IncFII, and IncFIB-I2 (Malhotra-Kumar et al., 2016; McGann et al., 2016; Poirel et al., 2016; Sun et al., 2016; Xavier et al., 2016; Zurfluh et al., 2016; Donà et al., 2017; Wang et al., 2017; Zhang et al., 2017). This was consistent with multiple *mcr-1* mobilization events which tend to potentially facilitate the association of *mcr-1* with other resistance mechanisms, thereby creating multidrug-resistant bacterial hosts (Zhong et al., 2018). Of these plasmids, IncI2-, IncX4-, and IncHI2-type ones are the most common vectors of *mcr-1*, with the insertion sequence (IS) IS*Apl1* mostly appearing up- or downstream of the gene (Matamoros, 2017). Few studies reported *mcr-1*-carrying hybrid plasmid and their mechanism of resistance. The hybrid plasmid IncX3-X4 is based on homologous recombination or the transposase-mediated formation of a cointegrate between IS*26* and the *nic* site of *oriT*. This also suggested that even if the insert sequence on the plasmid carrying the *mcr-1* gene does not mobilize the transfer of *mcr-1*, it can still be recombined with other plasmids containing the same insert sequence to form a more complex hybrid plasmid (Sun et al., 2016). These researches indicated that IS may contribute to plasmid-plasmid and plasmid-chromosome rearrangements as well as gene mobilization. Therefore, it might be significantly valuable to study IS on plasmids carrying the *mcr-1* gene.

This study aimed to investigate the population structure of MPECs and the features of *mcr-1*-carrying plasmids in MPECs from animals which showed reduced effectiveness to antimicrobial. The present study may improve the understanding of the epidemiology, likelihood of spread and the potential, and evolutionary mechanisms of the *mcr-1* gene in veterinary medicine.

## Materials and Methods

### Isolation of *mcr-1*-Positive *E. coli* Strains

Samples were obtained from lesion organs of swine, poultry (chicken), and bovine (dairy cow) between 2016 and 2019 in Yangling, Shaanxi, China. A total of 109 samples were collected from the organs with lesions in swine (*n* = 37) and chickens (*n* = 53) suffering from colibacillosis and the uterine rinsing fluid of dairy cows (*n* = 19) suffering from endometritis. The samples were collected in different farms, and each sample was from a different animal. The samples were stored aseptically at 4°C and transported to the laboratory in time for isolation and identification of strains. All animal procedures and study design were approved by the animal ethics committee of Northwest A&F University (Approval No.: 2016012).

All samples were cultivated on MacConkey agar (MAC) plates containing 2 μg/ml colistin and incubated at 37°C for 18 h. The species were further identified by the amplification and sequencing of 16S rRNA. Then, MPECs were detected by amplification and sequencing of *mcr-1* (Liu et al., 2016).

### Antimicrobial Susceptibility Testing of MPECs

Antimicrobial susceptibility testing was performed by broth microdilution method, as internationally recognized clinical guidelines those provided by the Clinical and Laboratory Standards Institute (CLSI) (CLSI, 2013, 2019). Briefly, 0.5 McFarland inoculum suspensions were diluted 1:100 to a final inoculum density of 10^5^ CFU/ml. Ampicillin (AMP), ceftazidime (CAZ), cefotaxime (CTX), aztreonam (AZM), tetracycline (TET), doxycycline (DOX), ciprofloxacin (CIP), enrofloxacin (ENR), gentamicin (GEN), sulfamethoxazole (SMZ), fosfomycin (FOS), florfenicol (FFC), colistin (COL), and meropenem (MEM) (Solarbio, Beijing, China) were selected to determine the minimum inhibitory concentrations (MICs) of MPECs. The antimicrobials were twofold serially diluted ranging from 0.25 to 512 μg/ml and incubated at 37°C for 18 h. The MICs were recorded as the lowest concentrations of antimicrobials in the wells, where no visible bacteria growth was observed.

### Multilocus Sequence Typing of MPECs

MPECs were sequenced using Illumina HiSeq TM2000 platform at the Novogene Bioinformatics (Beijing, China). For Illumina sequencing, at least 1 μg genomic DNA was used for each strain in sequencing library construction. DNA samples were sheared into 400–500 bp fragments using a Covaris M220 Focused Acoustic Shearer following manufacture’s protocol. Illumina sequencing libraries were prepared from the sheared fragments using the NextFlex^TM^ Rapid DNA-Seq Kit. The prepared libraries then were used for paired-end Illumina sequencing (2 × 150 bp) on an Illumina HiSeq TM2000 machine. The raw data generated from Illumina platform were submitted to Enterobase^1^, and sequence types were automatically assigned according to the Achtman scheme. A minimum spanning tree was generated using GrapeTree software to analyze the distribution of sequence type of MPECs (Zhou et al., 2018) characterized by multilocus sequence typing (MLST) analysis.

### Mating Experiment of MPECs

To evaluate the transferability of *mcr-1* gene, broth-mating experiments were performed with six representative MPECs (donor) and streptomycin-resistant *E. coli* C600 (recipient). Briefly, donor strain was mixed with recipient strain in LB broth (1:3) and the mixture was incubated at 37°C for 4 h in a shaker incubator (180 r/min). Subsequently, mixture was spread on MAC plates containing 2,000 μg/ml streptomycin (Solarbio, Beijing, China) and 2 μg/ml colistin and incubated at 37°C for 24 h to select transconjugants. The susceptibility of transconjugants for antimicrobials was determined based on the MICs.

### Whole-Genome Sequencing Analysis of MPECs

The total genomic DNA was extracted from representative MPECs with TIANamp Bacteria DNA Kit (Tiangen, Beijing, China) following the manufacturer’s instructions and sequenced (MiSeq, Illumina, San Diego, CA, United States), producing 2 × 150-bp paired-end reads (Majorbio Company, Shanghai, China) and long-read (Pacific Biosciences, Menlo Park, CA, United States) technology. The short and long reads were assembled using Unicycler (Wick et al., 2017). Gene prediction and annotation were done with RAST genome annotation server version 2.0^2^ and BLASTp. Plasmid replicon types were identified by the PlasmidFinder 2.1^3^ from the Center for Genomic Epidemiology (CGE). Acquired resistance genes and chromosomal mutation-mediated antimicrobial resistance genes (ARGs) were identified with ResFinder 4.1^4^ from CGE and resistance gene identifier (RGI)^5^ from the Comprehensive Antibiotic Resistance Database (CARD). Conjugal transfer components and type IV secretion systems (T4SS) of the plasmids were performed using oriTfinder^6^. Insertion sequence (IS) elements, transposon (Tn), and integron (In) were identified using ISfinder^7^, INTEGRALL^8^, and MobileElement Finder v1.0.3^9^. Virulence genes and FimH type were identified with VirulenceFinder 2.0^10^ and FimTyper 1.0^11^, respectively, available at the Center for Genomic Epidemiology. Phylogroups were predicted using the ClermonTyping tool at the IAME Research Center web^12^.

### Genetic Analysis of *mcr-1*-Carrying Plasmids

The genetic environment of *mcr-1*-carrying plasmid was compared with previously reported ones in GenBank with BLAST Ring Image Generator (BRIG) and EasyFig tools (v2.3) (Alikhan et al., 2011; Sullivan et al., 2011).

### Nucleotide Sequence Accession Numbers

The complete genomes were deposited in GenBank with a Accession Nos. CP047002 (J-8 chromosome), CP046418 (pS4-mcr-1.1), CP047014 (pmcr-1), CP059836 (pMCR_A3_2), and BioProject Accession No. PRJNA649870 (pMCR_J9_7).

## Results

### Antimicrobial Susceptibility Profiles of 24 MPECs

**FIGURE 1:**
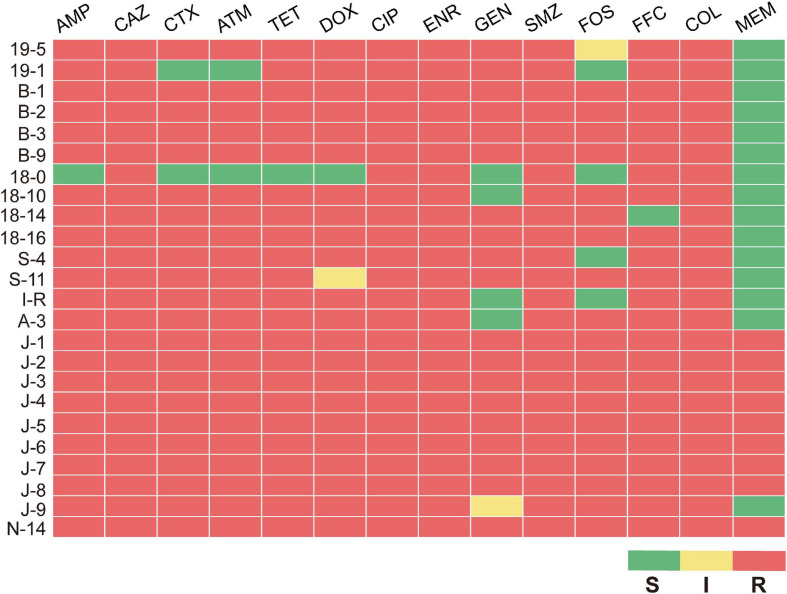
Antimicrobial sus ceptibility profile of 24 MPECs. S, susceptible; I, intermediate; R, resistant.

After isolating bacterial strains from 109 samples, 24 MPECs were screened based on *mcr-1* gene amplification and sequencing (data not shown). Among the 24 MPECs, nine strains were from poultry, 14 strains were from swine, and one strain was from bovine (Supplementary Table 2). The MICs of the 24 MPECs against 14 antimicrobials are as shown in Supplementary Table 1. All of MPECs were resistant to at least three or more antimicrobial categories; nine of 24 MPECs, namely J-1, J-2, J-3, J-4, J-5, J-6, J-7, J-8, and N-14, were resistant to all the 14 antimicrobials tested, including β-lactams (penicillins, cephems, monobactams, carbapenems), tetracyclines, fluoroquinolones, aminoglycosides, sulfonamides, phenicols, fosfomycins, and polymyxins. In addition, five of these (B-1, B-2, B-3, B-9, and18-16) were also sensitive only to meropenem (Figure 1). The above results indicated that all the 24 MPECs were resistant to at least three or all antimicrobial categories and exhibited either MDR or XDR for the common antimicrobials in *Enterobacteriaceae*.

### Genotypic Diversity of 24 MPECs

**FIGURE 2:**
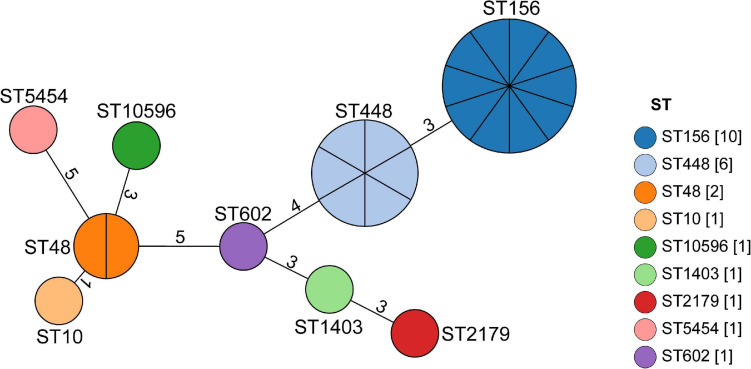
Minimum spanning tree analysis of the 24 MPECs. Each node represents a distinct sequence type (ST). The size of the circle represents the number of isolates with the same ST profile. The number on the line represents the count of the locus variants for ST.

Nine STs, namely ST156 (41.6%, 10/24), ST448 (25%, 6/24), ST48 (8.3%, 2/24), ST10 (4.2%, 1/24), ST10596 (4.2%,1/24), ST1403 (4.2%, 1/24), ST2179 (4.2%, 1/24), ST5454 (4.2%, 1/24), and ST602 (4.2%, 1/24) were identified from the 24 MPECs using MLST method (Figure 2). After analyzing the EnteroBase database (Supplementary Table 2), nine STs were further assigned to six distinct clonal complexes (CCs) (CC156, CC448, CC10, CC278, CC206, CC446) and two singletons (ST10596, ST2179). MLST analysis indicated the genetic diversity among 24 MPECs, and the dominant clones belonged to CC156, CC448, and CC10. Moreover, ST15096 was discovered from swine for the first time. These suggests that the number of *mcr-1*-carrying clonal complex is still increasing.

### Conjugative Transfer of *mcr-1* Gene in Six MPECs

Based on the antimicrobial resistance spectrum and the ST distribution of the 24 MPECs, six MPECs (S-4, A-3, J-8, J-9, 19-5, and B-2) were selected for the mating assay. Transconjugants were obtained from all donors except J-8 and B-2. These transconjugants exhibited the same level of colistin resistance (4 μg/ml) as the donors, but at the same time, both showed a 16-fold increase in resistance when compared with the recipient *E. coli* C600 (0.25 μg/ml) (Table 1). In the case of three *E. coli* transconjugants (Tc-S-4, Tc-J-9, and Tc-19-5), no additional resistance phenotype was cotransferred along with the *mcr-1* gene. However, for Tc-A-3, showed β-lactam, tetracycline, fluoroquinolone, sulfonamide, fosfomycin, and florfenicol resistance phenotypes were also observed in addition to that of colistin resistance. The results suggested that *mcr-1* genes of S-4, A-3, J-9, and 19–5 were located on the conjugative transfer plasmid and could be successfully transferred to a recipient strain to confer the colistin resistance phenotype. Although the *mcr-1* gene of both J-8 and B-2 conjugated failure, J-8 was resistant to all tested antimicrobials. Therefore, this strain was chosen, along with the other successfully conjugated ones for the next experiment.

**TABLE 1:** The MICs for MPECs and transconjugants.

Strain	MIC (μ g/mL)													
	COL	AMP	CAZ	CTX	AZM	TET	DOX	CIP	ENR	GEN	SMZ	FOS	FFC	MEM
EC600	<0.25	2	<0.25	<0.25	<0.25	2	1	<0.25	<0.25	1	32	64	2	<0.25
S-4	**4**	**>512**	**512**	**>512**	**128**	**512**	**64**	**256**	**256**	**128**	**>512**	64	**>512**	<0.25
Tc^1^-S-4	**4**	2	<0.25	<0.25	<0.25	<0.25	0.5	<0.25	<0.25	<0.25	32	64	2	<0.25
A-3	**4**	**>512**	**16**	**>512**	**512**	**256**	**64**	**256**	**4**	2	**>512**	**>512**	**>512**	<0.25
Tc^1^-A-3	**4**	**>512**	2	**>512**	**512**	**64**	**32**	**8**	**2**	1	**>512**	**>512**	**>512**	<0.25
J-9	**4**	**>512**	**>512**	**>512**	**>512**	**256**	**32**	**32**	**256**	8	**>512**	**>512**	**>512**	<0.25
Tc^1^-J-9	**4**	2	0.25	<0.25	<0.25	<0.25	0.25	<0.25	1	1	32	64	2	<0.25
19-5	**4**	**512**	**>512**	**16**	**16**	**512**	**32**	**64**	**128**	**256**	**>512**	128	**>512**	<0.25
Tc^1^-19-5	**4**	2	0.25	<0.25	<0.25	<0.25	0.25	<0.25	1	1	32	64	2	<0.25
J-8	**4**	**>512**	**>512**	**>512**	**256**	**128**	**16**	**128**	**256**	**256**	**>512**	**>512**	**512**	**64**
Tc^1^-J-8	-	-	-	-	-	-	-	-	-	-	-	-	-	-
B-2	**4**	**>512**	**>512**	**>512**	**512**	**256**	**32**	**128**	**128**	**128**	**>512**	**>512**	**16**	<0.25
Tc^1^-B-2	-	-	-	-	-	-	-	-	-	-	-	-	-	-

*^1^Tc represents the transconjugant; “-,” not detected; Bold, resistant.*

### Features of Resistance Genes and Plasmids in Five MPECs

**FIGURE 3:**
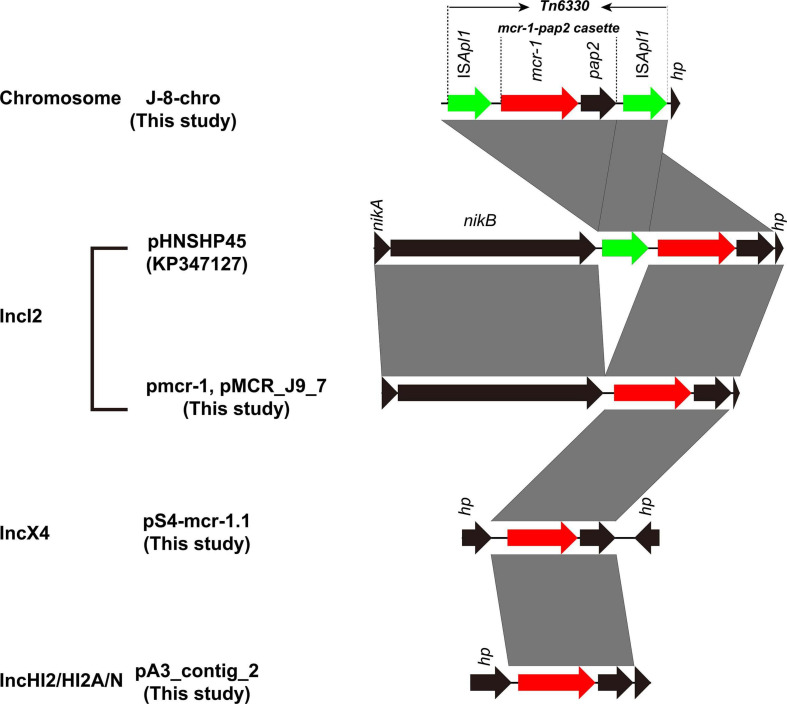
Genetic context of *mcr-1* gene in chromosome and plasmids from this study compared with plasmid pHNSHP45. Black arrows represent *nikA* (plasmid mobilization relaxosome protein), *nikB* (relaxase), and hypothetical protein (*hp*). Green arrows denote insertion sequence IS*Apl1*. Red arow denotes colistin resistance gene *mcr-1*. Regions of >99% identity are marked by gray shading.

Five MPECs (S-4, A-3, J-8, J-9, and 19–5) were selected for whole-genome sequencing (WGS) analysis to identify the gene and plasmid features which are responsible for resistance. Beside the *mcr-1* gene, all the five MPECs contained more than three different types of acquired resistance genes as well as at least two resistant plasmids (Table 2). In addition, locus mutations of *gyrA* (S83L, D87N) and *parC* (S80I) were also found on the chromosome. In the case of J-8, the *mcr-1* gene was located on the chromosome and flanked by two IS*Apl1* (Figure 3), with an additional *bla*_NDM–__5_ gene located on the IncX3-type plasmid. Conversely, the *mcr-1* genes of the other four MPECs were located on three different transferable plasmids IncHI2/HI2A/N (A-3), IncX4 (S-4), and IncI2 (J-9 and 19–5).

**TABLE 2:** Genomic features of the five representative MPECs.

Isolate name	FimH type	Phylogroup	Chromosome/Plasmid	Size (bp)	Inc type(s)	Resistance gene/Chromosomal point mutation	Virulence gene
A-3	*fimH25*	B1	chr-A-3_1	5,081,811		*mdf(A), gyrA* (S83L)	*gad, hra, lpfA, terC*
			pMCR_A3_2	267,184	HI2, HI2A, N	*mcr-1, bla*_OXA–__1_, *tet(A), tet(M), aadA1, aadA2, aph(3′)-Ia, aac(6′)-Ib-cr, oqxA, oqxB, qnrS2,sul1, sul3, dfrA12, mph(A), mef(B),floR, cmlA1, catB3, arr-3, qacE*Δ*1, qacH2*	*terC*
			pCTX_A3_3	133,615	I1-I(α)	*bla*_CTX–M–__65_, *aph(6)-Id, aph(3′′)-Ib, aph(3′)-Ia, fosA3*	*cib*
			pTET_A3_4	51,601	X1	*tet(A)*	-
			p0A3_5	82,387	FIB, FIC(FII)	-	*etsC*
S-4	-	A	chr-S-4_1	4,734,316		*tet(A), mdf(A), gyrA* (S83L, D87N)	*gad, papC, terC*
			pS4-aph	113,995	FIB	*bla*_TEM–__1__B_, *tet(M), aph(4)-Ia, aac(3)-IV, sul3*	*traT*
			pS4-mcr-1.1	34,645	X4	*mcr-1*	-
J-8	*fimH1127*	B1	chr-J-8_1	4,834,034		*mcr-1.1, bla*_TEM–__1__B_, *mdf(A), gyrA* (S83L, D87Y)	*astA, gad, iss, lpfA, papC, terC*
			pTEM	116,304	FIB, FIC(FII)	*bla*_TEM–__1__B_, *aadA2, aac(3)-IId, aph(3′)-Ia, oqxA, oqxB, dfrA12, sitABCD, mph(A)*	*cma, cvaC, hlyF, iroN, iss, iucC, iutA, ompT, sitA*
			pCTX	112,016	FIB	*blaCTX-M-55*	-
			pfosA3	88,744	Y	*aph(6)-Id, aph(3′′)-Ib, tet(A), sul2, floR, fosA3*	-
			pNDM	46,161	X3	*bla*_NDM–__5_	-
J-9	*fimH86*	B1	chr-J-9_1	4,948,775		*tet(A), aadA1, aph(3′′)-Ib, aph(6)-Id, sul2, dfrA1, floR, fosA7, mdf(A), gyrA* (S83L, D87N)	*gad, hra, lpfA, papA_F19, terC*
			pOQX_J9_2	140,375	FIB, FIC(FII)	*aph(3′)-Ia, oqxA, oqxB, dfrA17, sitABCD*	*cvaC, etsC, hlyF, iroN, iss, iucC, iutA, mchF, ompT, sitA, traT*
			pCTX_J9_3	101,266	FII, N	*bla*_TEM–__1__B_, *blaCTX-M-55, fosA3*	*traT*
			pMCR_J9_7	60,959	I2	*mcr-1*	-
19-5	*fimH54*	A	chr-19-5_1	4,710,580		*mdf(A), gyrA* (S83L, D87N), *parC* (S80I)	*gad, terC*
			pfloR	161,471	FIA, FIB, FII, Col156	*bla*_TEM–__1__B_, *aph(3′)-Ia, aac(3)-IId, tet(A), tet(M), sul2, sul3, aadA1, aadA2, aadA22, erm(42), floR, cmlA1, mph(A), sitABCD*	*celb, iucC, iutA, sitA, traT*
			pCMY-2	101,644	I1-I(α)	*bla*_CMY–__2_	*cib*
			p19-5_4	93,419	Unknown	-	-
			pmcr-1	60,734	I2	*mcr-1*	-
			pX4	46,955	X4	-	-
			pQ1	6,477	Q1	-	-

*chr-, chromosome; p-, plasmid; Inc-, incompatibility. Acquired resistance genes: colistin: *mcr-1*; β-lactam: *bla*_*TEM–*__1__*B*_, *bla*_*OXA–*__1_, *bla*_*CMY–*__2_, *bla*_*CTX–M–*__55_, *bla*_*CTX–M–*__65_, *bla*_*NDM–*__5_; tetracycline: *mdf(A)*, *tet(A)*, *tet(M)*; aminoglycosides: *aadA1*, *aadA2*, *aadA22*, *aph(3′)-Ia*, *aph(3′′)-Ib*, *aph(4)-Ia*, *aph(6)-Id*, *aac(3)-IId*, *aac(3)-IV*, *aac(6′)-Ib-cr*; fluoroquinolones: *gyrA* (point mutations: S83L, TCG→TTG; D87N, GAC→AAC), *parC* (point mutation: S80I, AGC→ATC), *aac(6′)-Ib-cr*, *oqxA*, *oqxB*, *qnrS2*; macrolides: *mdf(A)*, *mph(A), mef(B)*, *erm(42)*; sulfonamides/trimethoprim: *sul1, sul2*, *sul3*, *dfrA1*, *dfrA12*, *dfrA17*; phenicol: *catB3*, *cmlA1*, *floR*; fosfomycin: *fosA3*, *fosA7*; rifamycin: *arr-3*; quaternary ammonium compound: *qacE*Δ*1*, *qacH2*; peroxides: *sitABCD*. All of the five MPECs mainly belonged to phylogroup A and B1 and subtyping of the *fimH* gene showed four *fimH*-type (*fimH25*, *fimH1127*, *fimH86*, and *fimH54*). Virulence genes were mainly located on IncF-type plasmids and chromosomes.*

### Genomic Features of *mcr-1*-Carrying Plasmids

**FIGURE 4:**
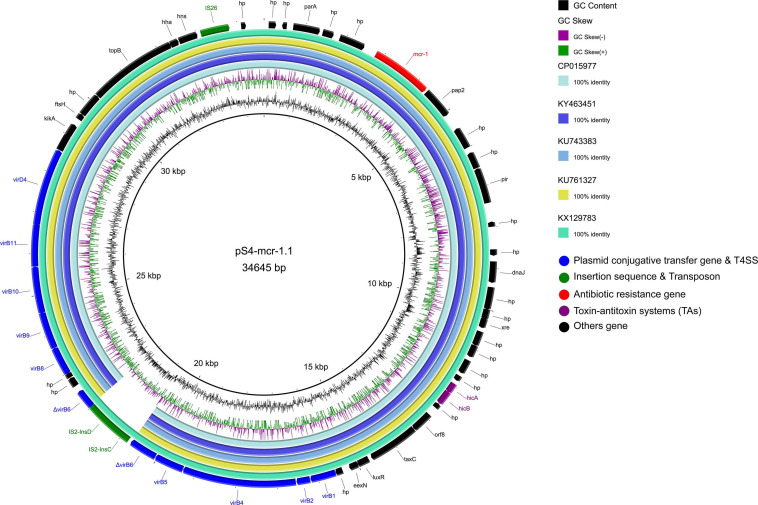
Comparative genomic analysis of *mcr-1*-carrying IncX4-type plasmid pS4-mcr-1.1 (this study) with plasmids pICBEC72Hmcr (CP015977), pOW3E1 (KX129783), pMCR_WCHEC1606 (KY463451), pESTMCR (KU743383), and pmcr1_IncX4 (KU761327) in GenBank. The plasmid pS4-mcr-1.1 was used as a reference sequence. Dark gray shading denotes regions of homology (>99% identity nucleotide identity).

**FIGURE 5:**
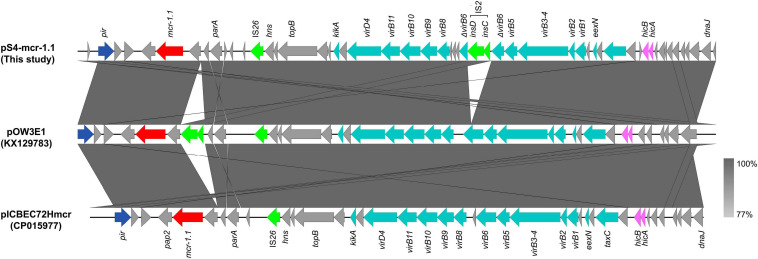
Colinear sequence comparison of IncX4-type *mcr-1*-carrying plasmid pS4-mcr-1.1 (this study) with other plasmids including pOW3E1 (KX129783) and pICBEC72Hmcr (CP015977). Boxed arrows represent the position and transcriptional direction of ORFs. Dark gray shading denotes regions of homology (>99% identity nucleotide identity). Blue colors represent the plasmid replication- and maintenance/stability-associated genes; teal, plasmid conjugative transfer genes and T4SS genes; green, IS and Tn; red, antimicrobial resistance genes; pink, toxin-antitoxin system (TAs) genes; gray, other genes.

The *mcr-1* gene in strain S-4, located on the plasmid pS4-mcr-1.1 (34,645 bp; GC content of 42.25%), belonged to the IncX4 incompatibility group (Table 2). The backbone of this plasmid was highly similar to those of pICBEC72Hmcr (CP015977; nucleotide coverage 96%; identity 99.98%), pOW3E1 (KX129783; nucleotide coverage 100%; identity 99.59%), pMCR_WCHEC1606 (KY463451; nucleotide coverage 96%; identity 99.73%), pESTMCR (KU743383; nucleotide coverage 96%; identity 99.58%), and pmcr1_IncX4 (KU761327; nucleotide coverage 96%; identity 99.44%) as indicated by BLASTn analysis (Figure 4). The backbone structure of all those six plasmids was highly conserved, with the only difference being that the *virB6* gene of the T4SS system on the IncX4-type plasmid was truncated by an IS*2*. From these plasmids, two, namely, pOW3E1 and pICBEC72Hmcr, were selected for collinear alignment with pS4-mcr-1.1. The results indicated that no resistance genes other than that of *mcr-1* was identified, with the gene embedded in an *mcr-1-pap2* cassette of approximately 2.6 kb (Figures 3, 5). Furthermore, even though all plasmids contained an insert sequence IS*26*, the insertion sequence IS*Apl1* was absent either upstream or downstream of the *mcr-1* gene. Besides, on pS4-mcr-1.1, *virB6* gene in T4SS was truncated into two fragments by IS*2*. In the case of pOW3E1, IS*2* was found upstream of the *mcr-1* gene. In addition, a type II toxin-antitoxin system (TAs) *hicA/hicB* was also found between upstream of T4SS and downstream of the chaperone-encoding *dnaJ*.

**FIGURE 6:**
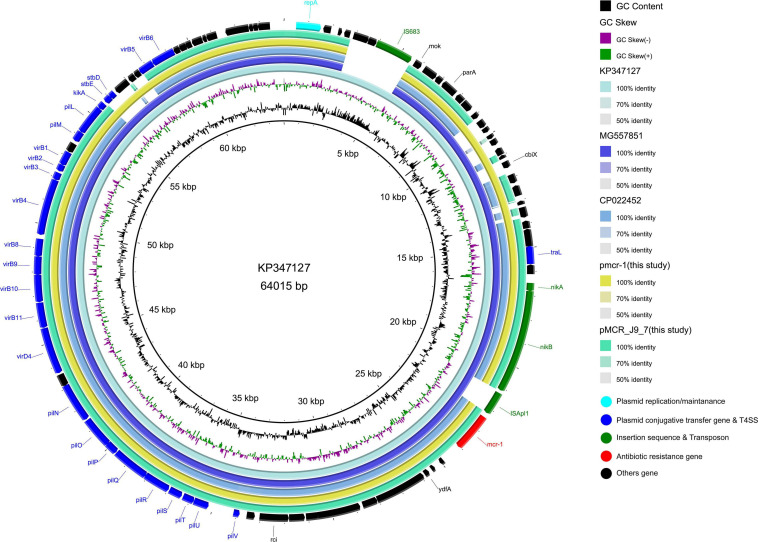
Comparative genomic analysis of *mcr-1*-bearing IncI2-type plasmids pmcr-1 (this study) and pMCR_J9_7 (this study) compared with plasmids pD90-2 (CP022452), pHNSHP45 (KP347127), and PN21 (MG557851) in GenBank. The plasmid pHNSHP45 was used as a reference sequence.

**FIGURE 7:**
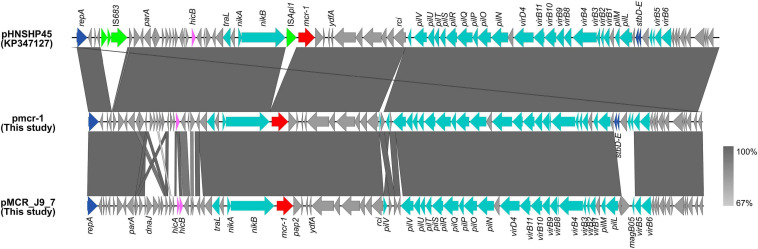
Colinear sequence comparison of IncI2 reference plasmid pHNSHP45 (GenBank accession No. KP347127) with plasmids including pmcr-1 (this study) and pMCR_J9_7 (this study). Boxed arrows represent the position and transcriptional direction of ORFs. Dark gray shading denotes regions of homology (>99% identity nucleotide identity). Blue colors represent the plasmid replication- and maintenance/stability-associated genes; teal, plasmid conjugative transfer genes and T4SS genes; green, IS and Tn; red, antimicrobial resistance genes; pink, TAs genes; gray, other genes.

The *mcr-1* in strain 19-5 and J-9 was, respectively, carried by the plasmid pmcr-1 (60,734 bp; GC content of 42.50%) and the plasmid pMCR_J9_7 (60,959 bp; GC content of 42.36%), all belonging to the IncI2 incompatibility group (Table 2). BLASTn analysis showed that pMCR_J9_7 was similar to the plasmid pD90-2 (CP022452; nucleotide coverage 99%; identity 99.99%) and pHNSHP45 (KP347127; nucleotide coverage 90%; identity 99.63%), while pmcr-1 was similar to the plasmid PN21 (MG557851; nucleotide coverage 100%; identity 99.97%) and pHNSHP45 (KP347127; nucleotide coverage 99%; identity 99.97%) (Figure 6). A sequence comparison of the two plasmids indicated that they shared 99.63% identity with 91% nucleotide coverage. The gaps shown in the circle diagram represented the differences between the five *mcr-1*-bearing plasmids and the absence of IS*683* and IS*Apl1*. For colinear analysis with the plasmids pMCR_J9_7 and pmcr-1, pHNSHP45 was selected as reference one. As shown in Figure 7, IS*Apl1* located upstream of the *mcr-1* gene and IS*683* located between *repA* and *parA* were absent in plasmid pMCR_J9_7 and pmcr-1. In addition, *stbD* and *stbE* genes, both of which are located downstream of *virB5* and encoding stability proteins, were absent from the plasmid pMCR_J9_7. A similar observation was made for *dnaJ*, a chaperone-encoding gene, which was absent between *parA* and *traL* in plasmid pmcr-1. For both identified IncI2-type plasmids, only the *mcr-1* gene, embedded within the *mcr-1-pap2* cassette, was associated with colistin resistance (Figure 3). Moreover, a type II toxin-antitoxin system (TAs) *hicA*/*hicB* was found to be present on the plasmid pMCR_J9_7, with the *hicA* gene, encoding antitoxin proteins, being absent from the plasmid pmcr-1.

**FIGURE 8:**
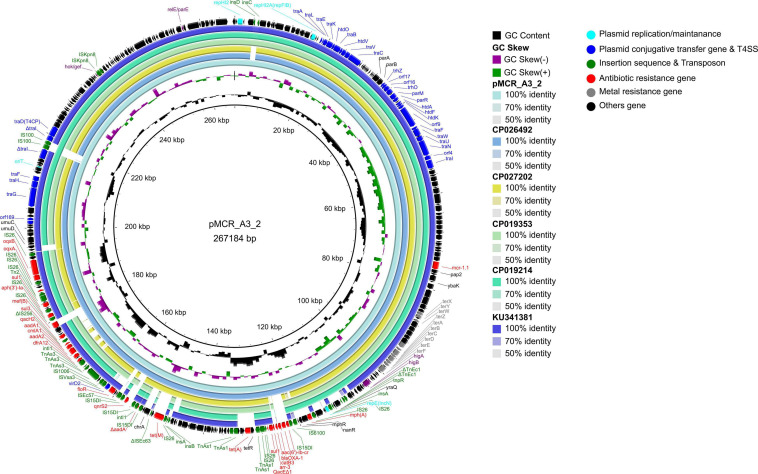
Comparative genomic analysis of *mcr-1*-bearing IncHI2-type plasmid pMCR_A3_2 (this study) compared with plasmids pHS13-1-IncHI2 (CP026492), pXH988_1 (CP019353), pMCR1_025943 (CP027202), pMCR_WCHEC050613 (CP019214), and pHNSHP45-2 (KU341381) in GenBank. The plasmid pHNSHP45-2 was used as a reference sequence.

**FIGURE 9:**
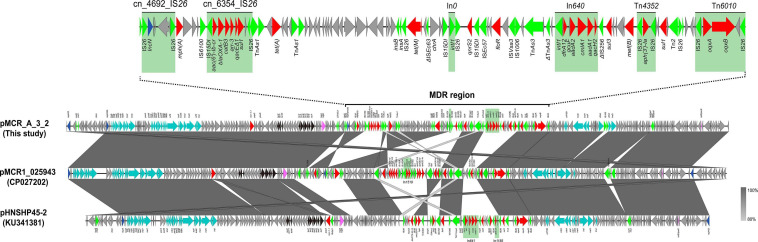
Colinear sequence comparison of IncHI2/HI2A/N plasmid pMCR_A3_2 (this study) with other plasmids including pMCR1_025943 (GenBank accession No. CP027202) and pHNSHP45-2 (GenBank accession No. KU341381). Boxed arrows represent the position and transcriptional direction of ORFs. Dark gray shading denotes regions of homology (>99% identity nucleotide identity). Blue colors represent the plasmid replication- and maintenance/stability-associated genes; teal, plasmid conjugative transfer (*tra*-/*trb*- gene cluster) and T4SS genes; green, IS and Tn; red, antimicrobial resistance genes; pink, TA genes; gray, other gene. The light green shade represents the composition transposon and integron (In); MDR, multidrug resistant.

The *mcr-1* gene in strain A-3 was carried by a large multireplicon MDR plasmid (designated pMCR_A3_2; 267,184 bp; GC content of 46.84%) containing IncHI2, IncHI2A, and IncN replicons (Table 2). Compared with pHNSHP45-2, a IncHI2-type reference plasmid, the plasmid pMCR_A3_2 and pMCR1_025943 lacked IS*Apl1* upstream of the *mcr-1* gene which was embedded in a 2.6-kb *mcr-1-pap2* gene cassette (Figure 3). The backbone of pMCR_A3_2 was highly similar to those of pHS13-1-IncHI2 (CP026492; nucleotide coverage 98%; identity 99.94%), pXH988_1 (CP019353; nucleotide coverage 94%; identity 99.99%), pMCR1_025943 (CP027202; nucleotide coverage 92%; identity 99.99%), pMCR_WCHEC050613 (CP019214; nucleotide coverage 92%; identity 99.99%), and pHNSHP45-2 (KU341381; nucleotide coverage 88%; identity 99.99% (Figure 8). The gaps in the circle diagram showed the differences between the six *mcr-1*-bearing plasmids, including the insertion or deletion of IS, Tn, and ARGs. In the case of this plasmid (pMCR_A3_2), pMCR1_025943 and pHNSHP45-2 were selected as reference plasmids for colinear analysis. As shown in Figure 9, the differences between pMCR_A3_2 and those three reference plasmids were mainly focused on the MDR region. For pMCR_A3_2, this region included two class 1 integrons, namely In*0* (no resistance gene cassette) and In*640* (*dfrA12*, *gcuF*, *aadA2*, *cmlA1*, *aadA1a*, *qacH2*) which confer resistance to trimethoprims, aminoglycosides, chloramphenicols, and quaternary ammonium compounds. Between these two integrons, the fluoroquinolone resistance gene *qnrS2* and the phenicol resistance gene *floR* were present. In addition, downstream of In*640*, there are two composite transposons, namely, Tn*4352* (IS*26*-*aph(3′)*-Ia-IS*26*) which carried an aminoglycoside resistance gene *aph(3′)-Ia* and Tn*6010* (IS*26*-*oqxA*-*oqxB*-IS*26*) which carried the fluoroquinolone resistance genes *oqxA* and *oqxB*. Between the In*640* and Tn*4352*, the macrolide efflux pump gene *mef(B)* and the sulfonamide resistance gene *sul3* were also present, along with another sulfonamide resistance gene *sul1* presented between these two composite transposons. The MDR region further contained two other transposons, namely, cn_4692_IS*26* (IS*26*-*repE*-*hp*-*hp*-*hp*-*hp*-IS*26*) and cn_6354_IS*26* [IS*15DI*-*aac(6′)*-Ib-cr-*bla*_OXA–__1_-*catB3*-*arr-3*-*qacE*Δ*1*-*sul1*-*hp*-IS *26*], and the latter transposon cn_6354_IS*26* conferred resistance to aminoglycoside/fluoroquinolone, beta-lactam, phenicol, rifamycin, quaternary ammonium compounds, and sulfonamide. An additional macrolide resistance gene *mph(A)* as well as the tetracycline resistance genes *tet(A)* and *tet(M)* and the tellurite resistance operon were also presented on pMCR_A3_2. Insertion sequences and complex transposons such as IS*6* family (IS*1006*, IS*6100*), Tn*3* family (Tn*As1*, Tn*As3*, Tn*Ec1*, Tn*Ec3*, Tn*Ec63*, Tn*2*), IS*21* family (IS*Ec57*, IS*100*), IS*91* family (IS*Vsa3*), IS*3* family (IS*Kpn8*), and IS*256* family (IS*256*) were found on the plasmids as well. Eventually, a relaxase-encoding *traI* gene in T4SS system was found to be truncated into two fragments by IS*100*. Regarding the presence of toxin-antitoxin systems (TAs), three type II ones, namely, *higA/higB*, *hok/gef*, and *relE/parE* were located on the pMCR_A_3_2.

## Discussion

MPECs from different sources have been isolated worldwide. In the present study, 24 MPECs were isolated from organs having lesions obtained from animals, which did not respond to antimicrobial treatment and which were, therefore, considered to show MDR, or even XDR based on the definition provided by Magiorakos et al. (2012). MLST analysis classified the 24 MPECs into nine sequence types (six dominant CCs and two singletons), among which ST10596 has been identified for the first time. The main CCs were CC156 (ST156), CC448 (ST448), and CC10 (ST48 and ST10).

MPECs of CC156 has been previously isolated only from the patients’ blood culture and ducks’ rectal swab (Yang et al., 2016; Rossi et al., 2017; Lin et al., 2020), which exhibited MDR or XDR including meropenem and colistin. In this study, 10 such MPECs, which showed resistance to β-lactamases (penicillins, cephems, monobactams, carbapenems), tetracyclines, fluoroquinolones, aminoglycosides, sulfonamides, phenicols, fosfomycins, and polymyxins, were recovered from chickens and bovines. In the case of MPEC ST156 (J-8), *mcr-1*, *bla_TEM–__1__B_, bla_CTX–M–__55_, bla*_NDM–__5_, *mdf(A), aadA2, aac(3)-IId, aph(3′)-Ia, aph(6)-Id, aph(3′′)-Ib, oqxA, oqxB, mph(A), tet(A), sul2, dfrA12, floR*, and *fosA3* genes were found to contribute to the resistance phenotype.

*Escherichia coli* ST10 and ST48 (single locus variant of ST10), belonging to CC10, are considered widely disseminated clones worldwide. CC10 is highly disseminated *via* foods, the environment, animals, and humans (Matamoros, 2017), and they are usually associated with the presence of colistin (*mcr-1*) (Papa-Ezdra et al., 2020), ESBL (*bla*_CTX–M–__14__/–__15_), and fluroquinolone [*aac(6′)-Ib-cr* and *qnrB*] (Oteo et al., 2009; Fam et al., 2011) resistance genes. However, in this study, in addition to those genes, MPEC ST10 (S-4) and 19-5 (ST48) were also found to contain aminoglycoside, tetracycline, sulfonamide, macrolide, lincosamide, and phenicol, as well as peroxides resistance genes. These results suggested that CC10 possessed a more extensive set of resistance genes and it could, therefore, become a reservoir for the transmission of resistance, and CC156 also could be a reservoir for *mcr-1* genes in a similar way to CC10. Furthermore, MPECs of ST448 are generally sporadic and not dominant (Bai et al., 2018), but results showed that six MPECs of ST448 were members of CC448, hence suggesting that CC448 could become a new dominant clone complex carrying the *mcr-1* gene in pig farms.

The *mcr-1* gene along with other resistance genes are found on conjugative transfer plasmids or mobile element transposons. As such, there is a risk for the horizontal transmission of colistin as well as other drug resistance between bacteria in the infected guts of animals, thereby further exacerbating the risk of spreading resistance (Guenther et al., 2017; Bai et al., 2018; Li et al., 2020). The results of this study showed that the *mcr-1* genes of strains S-4, A-3, J-9, and 19-5 located on the three different transferable plasmids IncHI2/HI2A/N (A-3), IncX4 (S-4), and IncI2 (J-9, 19-5), could be successfully transferred. Despite the fact that IncI2-, IncX4-, and IncHI2-type plasmids were the most common carriers of the *mcr-1* gene, yet their mobile elements (IS, Tn, In) were very different. Therefore, the study and description of mobile elements on plasmids can greatly contribute to the understanding of the coupling transfer of plasmids, the formation of hybrid plasmids, and the translocation of ARGs.

Self-transmissible IncX4-type plasmid, a key vector for the transmission of the *mcr-1* gene between *Enterobacteriaceae* worldwide, is the most common one which carries the resistance gene due to its high conjugative transfer ability (Biswas et al., 2020; Migura-Garcia et al., 2020). The structure of *mcr-1*-carrying IncX4-type plasmids are highly conserved, and they consist of *mcr-1*-*pap2* gene cassette, T4SS systems, plasmid replication/maintenance genes, TA modules, and an insertion sequence IS*26* (Bai et al., 2018). The IncX4-type plasmid pOW3E1, in addition to the previously mentioned modules, contained IS*2* close to the *mcr-1* gene (Zurfluh et al., 2016), but the insertion sequence had no effect on either the gene or the conjugative transfer of plasmid. In the present study, IS*26* and IS*2* were found on the IncX4-type plasmid pS4-mcr-1.1, with IS2 truncating the *virB6* gene of T4SS. Since the *virB6* product contributes to DNA transfer as a component of the endosomal translocation channel protein (Álvarez-Rodríguez et al., 2020), its truncation may affect the efficiency of plasmid conjugative transfer, although the process through which this occurs remains unknown.

The first reported *mcr-1*-carrying IncI2-type plasmid pHNSHP45 was identified in *E. coli* of swine origin in China and IS*Apl1* was located upstream of the resistance gene (Liu et al., 2016). However, in this study, the two IncI2-type plasmids (pmcr-1 and pMCR_J9_7) did not have any IS*Apl1* adjunct to the *mcr-1* gene, with IS*683* also lacking between *repA* and *parA*. Furthermore, colinear analysis of the plasmid pHNSHP45 along with those of pMCR_J9_7 and pmcr-1 indicated the absence of *stbD* and *stbE* genes, both encoding plasmid stability proteins, from pMCR_J9_7. Similarly, the *dnaJ* gene, encoding for the chaperone protein, was absent from the pmcr-1 plasmid. These results implied that the IncI2-type plasmid could lose some insertion sequences during the evolution process to maintain the conservative plasmid backbone structure, which is conducive to the stability of the *mcr-1* gene and the conjugative transfer of the plasmid.

IncHI2/HIA-type plasmids are usually > 250 kb, unlike IncX4- and IncI2-type plasmids. In addition, they contain an MDR region with various ISs, transposons, and integrons that capture various exogenous resistance genes, thereby conferring multidrug resistance and metal tolerance to *E. coli* (Fang et al., 2016). However, despite these differences, the backbone of IncHI2/HI2A-type plasmids carrying *mcr-1* gene are also conserved, in a similar way to those of the IncX4 and IncI2 plasmids. Hence, they were found to include plasmid replication/maintenance genes, T4SS systems, tellurium ion resistance gene clusters, and TA modules. Studies on the IncHI2-type plasmids carrying the *mcr-1* gene mostly focused on the description of the genetic environment of the gene, while the mobile elements of the MDR region of the IncHI2 type were overlooked. In this context, it is to be noted that the IncHI2/HI2A-type plasmid pEGY1-MCR-1 carrying the *mcr-1* gene, is flanked by two IS*Apl1* elements as well as In*641* (Hammad et al., 2019). In this study, pMCR_A3_2, a IncHI2/HI2A/N-type hybrid plasmid with an extra IncN-type replicon, was flanked by two IS*26* present in the MDR region. This implied that the replicon could have been transposed by a transposon composed of IS*26*. Alternatively, this could have been the result of homologous recombination or the transposase-mediated formation of a cointegrate from IS*26* and the *nic* site of *oriT*. Additional results showed that the MDR region of the pMCR_A3_2 plasmid, identified in this study, included two class 1 integrons, namely, In*0* and In*640*. However, that of the IncHI2-type plasmid pHNSHP45-2 carrying the *mcr-1* gene has been reported to contain two different class 1 integrons, namely, In*1185* and In*641* (Zhi et al., 2016). Furthermore, In*1519* was also identified from plasmid pMCR1_025943 (CP027202). Apart from those class 1 integrons, a large number of insertion sequences and transposons such as IS*26*/*26*-like (15*DI*) and Tn*3* families, were found to be present in the MDR region. The presence of these mobile integration elements facilitates the capture of numerous classes of resistance genes, thereby conferring various resistance phenotypes to *E. coli* containing the IncHI2-type plasmids.

Generally, IS*Apl1* is detected upstream of the *mcr-1* gene and is believed to be involved in the mobilization of the *mcr-1* cassette (Snesrud et al., 2016). In this study, the IS*Apl1* was absent from both upstream and downstream of the *mcr-1* gene on IncX4, IncI2, and IncHI2/HI2A/N plasmids, even though *mcr-1* on the chromosome was embedded in Tn*6330*. Previous studies have shown that the transposon Tn*6330* was immobilized on several plasmid backgrounds, following the loss of the flanking IS*Apl1* elements due to the high instability of being spread through plasmid transfer (Li et al., 2017; Wang et al., 2018). The loss of the IS*Apl1* transposon element in *mcr-1*-carrying plasmids has recently been proposed to be essential for the maintenance of the *mcr-1* gene. This is believed to be more beneficial to the host bacteria for adapting to environmental changes, especially when adapting from the pressure of antimicrobial agents to a pressure-free environment (Porse et al., 2016).

## Conclusion

In conclusion, 24 MPECs in our study showed MDR or even XDR and exhibited the population diversity. The T4SS genes located on IncX4- and IncHI2/HI2A/N-type plasmids were truncated by the insertion sequence. The truncation may affect the efficiency of plasmid conjugative transfer. Additionally, the MDR region of IncHI2/HI2A/N-type plasmids contained two class 1 integrons and four composite transposons. These mobile elements contributed to the multireplicon plasmid formation and the acquisition and transfer of ARGs.

## Data Availability Statement

The datasets presented in this study can be found in online repositories. The names of the repository/repositories and accession number(s) can be found in the article/Supplementary Material.

## Ethics Statement

The animal study was reviewed and approved by all animal procedures and study design were approved by the animal Ethics Committee of Northwest A&F University (Approval No: 2016012). Written informed consent was obtained from the owners for the participation of their animals in this study.

## Author Contributions

XS and ZL: conceptualization. ZL: methodology, software, formal analysis, data curation, and visualization. XS, YL, YF, and WM: validation. JL, HM, SL, BC, and HH: investigation. WZ: resources. ZL and YL: writing – original draft preparation. XS and YL: writing, review, and editing. XS: supervision, project administration, and funding acquisition. All authors have read and agreed to the published version of the manuscript.

## Conflict of Interest

The authors declare that the research was conducted in the absence of any commercial or financial relationships that could be construed as a potential conflict of interest.

## Publisher’s Note

All claims expressed in this article are solely those of the authors and do not necessarily represent those of their affiliated organizations, or those of the publisher, the editors and the reviewers. Any product that may be evaluated in this article, or claim that may be made by its manufacturer, is not guaranteed or endorsed by the publisher.

## Footnotes

1
https://enterobase.warwick.ac.uk/

2
https://rast.nmpdr.org/rast.cgi

3
https://cge.cbs.dtu.dk/services/PlasmidFinder/

4
https://cge.cbs.dtu.dk//services/ResFinder/

5
https://card.mcmaster.ca/analyze/rgi

6
https://tool-mml.sjtu.edu.cn/oriTfinder/oriTfinder.html

7
https://www-is.biotoul.fr/blast.php

8
http://integrall.bio.ua.pt/?search

9
https://cge.cbs.dtu.dk/services/MobileElementFinder/

10
https://cge.cbs.dtu.dk/services/VirulenceFinder/

11
https://cge.cbs.dtu.dk/services/FimTyper/

12
http://clermontyping.iame-research.center/

## References

[B1] Alikhan N. Petty N. K. Ben Zakour N. L. Beatson S. A. (2011). BLAST ring image generator (BRIG): simple prokaryote genome comparisons. *BMC Genomics* 12:402. 10.1186/1471-2164-12-402 21824423 PMC3163573

[B2] Álvarez-Rodríguez I. Arana L. Ugarte-Uribe B. Gómez-Rubio E. Martín-Santamaría S. Garbisu C. (2020). Type IV coupling proteins as potential targets to control the dissemination of antibiotic resistance. *Front. Mol. Biosci.* 7:201. 10.3389/fmolb.2020.00201 32903459 PMC7434980

[B3] Aslam B. Wang W. Arshad M. I. Khurshid M. Muzammil S. Rasool M. H. (2018). Antibiotic resistance: a rundown of a global crisis. *Infect*. *Drug*. *Resist*. 11 1645–1658. 10.2147/IDR.S173867 30349322 PMC6188119

[B4] Bai F. Li X. Niu B. Zhang Z. Malakar P. K. Liu H. (2018). A *mcr-1*-carrying conjugative IncX4 plasmid in colistin-resistant *Escherichia coli* ST278 strain isolated from dairy cow feces in Shanghai, China. *Front. Microbiol.* 9:2833. 10.3389/fmicb.2018.02833 30559724 PMC6287198

[B5] Biswas S. Elbediwi M. Gu G. Yue M. (2020). Genomic characterization of new variant of hydrogen sulfide (H_2_S)-producing *Escherichia coli* with multidrug resistance properties carrying the *mcr-1* gene in China^†^. *Antibiotics* 9:80. 10.3390/antibiotics9020080 32069849 PMC7167817

[B6] CLSI (2013). *Performance Standards for Antimicrobial Disk and Dilution Susceptibility Tests for Bacteria Isolated From Animals. Approved Standard-Fourth Edition.* CLSI document VET01-A4. Wayne, PA: Clinical and Laboratory Standards Institute, S10–S11.

[B7] CLSI (2019). *Performance Standards for Antimicrobial Susceptibility Testing.* CLSI supplement M100, 30th Edn. Wayne, PA: Clinical and Laboratory Standards Institute, 33–41.

[B8] Donà V. Bernasconi O. J. Pires J. Collaud A. Overesch G. Ramette A. (2017). Heterogeneous genetic location of *mcr-1* in colistin-resistant *Escherichia coli* isolates from humans and retail chicken meat in Switzerland: emergence of *mcr-1*-carrying IncK2 plasmids. *Antimicrob. Agents Chemother.* 61 e01245–17. 10.1128/AAC.01245-17 28848010 PMC5655086

[B9] Falgenhauer L. Waezsada S. Yao Y. Imirzalioglu C. Käsbohrer A. Roesler U. (2016). Colistin resistance gene *mcr-1* in extended-spectrum β-lactamase-producing and carbapenemase-producing gram-negative bacteria in Germany. *Lancet. Infect. Dis.* 16 282–283. 10.1016/S1473-3099(16)00009-8 26774242

[B10] Fam N. Leflon-Guibout V. Fouad S. Aboul-Fadl L. Marcon E. Desouky D. (2011). CTX-M-15-producing *Escherichia coli* clinical isolates in Cairo (Egypt), including isolates of clonal complex ST10 and clones ST131, ST73, and ST405 in both community and hospital settings. *Microb. Drug. Resist.* 17 67–73. 10.1089/mdr.2010.0063 21128836

[B11] Fang L. Li X. Li L. Li S. Liao X. Sun J. (2016). Co-spread of metal and antibiotic resistance within ST3-IncHI2 plasmids from *E. coli* isolates of food-producing animals. *Sci. Rep.* 6:25312. 10.1038/srep25312 27143648 PMC4855149

[B12] Guenther S. Falgenhauer L. Semmler T. Imirzalioglu C. Chakraborty T. Roesler U. (2017). Environmental emission of multiresistant *Escherichia coli* carrying the colistin resistance gene *mcr-1* from German swine farms. *J*. *Antimicrob*. *Chemother*. 72 1289–1292. 10.1093/jac/dkw585 28122910

[B13] Hammad A. M. Hoffmann M. Gonzalez-Escalona N. Abbas N. H. Yao K. Koenig S. (2019). Genomic features of colistin resistant *Escherichia coli* ST69 strain harboring *mcr-1* on IncHI2 plasmid from raw milk cheese in Egypt. *Infect. Genet. Evol.* 73 126–131. 10.1016/j.meegid.2019.04.021 31029792

[B14] Hordijk J. Farmakioti E. Smit L. A. M. Duim B. Graveland H. Theelen M. J. P. (2020). Fecal carriage of extended-spectrum-β-lactamase/AmpC-producing *Escherichia coli* in horses. *Appl. Environ. Microb.* 86 e02590–19. 10.1128/AEM.02590-19 32033947 PMC7117932

[B15] Kempf I. Jouy E. Chauvin C. (2016). Colistin use and colistin resistance in bacteria from animals. *Int. J. Antimicrob. Agents* 48 598–606. 10.1016/j.ijantimicag.2016.09.016 27836380

[B16] Li R. Xie M. Zhang J. Yang Z. Liu L. Liu X. (2017). Genetic characterization of *mcr-1*-bearing plasmids to depict molecular mechanisms underlying dissemination of the colistin resistance determinant. *J. Antimicrob. Chemother.* 72 393–401. 10.1093/jac/dkw411 28073961

[B17] Li X. Sun R. Song J. Fang L. Zhang R. Lian X. (2020). Within-host heterogeneity and flexibility of *mcr-1* transmission in chicken gut. *Int*. *J*. *Antimicrob*. *Agents* 55:105806. 10.1016/j.ijantimicag.2019.09.010 31533074

[B18] Lin Y. Yang L. Lu L. Wang K. Li J. Li P. (2020). Genomic features of an *Escherichia coli* ST156 strain harboring chromosome-located *mcr-1* and plasmid-mediated *bla*NDM-5. *Infect*. *Genet*. *Evol*. 85:104499. 10.1016/j.meegid.2020.104499 32791239

[B19] Liu Y. Y. Wang Y. Walsh T. R. Yi L. X. Zhang R. Spencer J. (2016). Emergence of plasmid-mediated colistin resistance mechanism MCR-1 in animals and human beings in China: a microbiological and molecular biological study. *Lancet. Infect. Dis*. 16:161. 10.1016/S1473-3099(15)00424-7 26603172

[B20] Luo J. Yao X. Lv L. Doi Y. Huang X. Huang S. (2017). Emergence of *mcr-1* in *Raoultella ornithinolytica* and *Escherichia coli* isolates from retail vegetables in China. *Antimicrob. Agents Chemother.* 61 e01139–17. 10.1128/AAC.01139-17 28739785 PMC5610531

[B21] Magiorakos A. P. Srinivasan A. Carey R. B. Carmeli Y. Falagas M. E. Giske C. G. (2012). Multidrug-resistant, extensively drug-resistant and pandrug-resistant bacteria: an international expert proposal for interim standard definitions for acquired resistance. *Clin*. *Microbiol*. *Infect*. 18 268–281. 10.1111/j.1469-0691.2011.03570.x 21793988

[B22] Malhotra-Kumar S. Xavier B. B. Das A. J. Lammens C. Butaye P. Goossens H. (2016). Colistin resistance gene *mcr-1* harboured on a multidrug resistant plasmid. *Lancet. Infect. Dis.* 16 283–284. 10.1016/S1473-3099(16)00012-8 26774247

[B23] Martin J. K. Sheehan J. P. Bratton B. P. Moore G. M. Mateus A. Li S. H. (2020). A dual-mechanism antibiotic kills gram-negative bacteria and avoids drug resistance. *Cell* 181 1518–1532. 10.1016/j.cell.2020.05.005 32497502 PMC7780349

[B24] Matamoros S. (2017). Global phylogenetic analysis of *Escherichia coli* and plasmids carrying the *mcr-1* gene indicates bacterial diversity but plasmid restriction. *Sci. Rep.* 7:15364. 10.1038/s41598-017-15539-7 29127343 PMC5681592

[B25] McGann P. Snesrud E. Maybank R. Corey B. Ong A. C. Clifford R. (2016). *Escherichia coli* harboring *mcr-1* and *bla*CTX-M on a novel IncF plasmid: first report of *mcr-1* in the United States. *Antimicrob. Agents Chemother.* 60 4420–4421. 10.1128/AAC.01103-16 27230792 PMC4914657

[B26] Migura-Garcia L. González-López J. J. Martinez-Urtaza J. Aguirre Sánchez J. R. Moreno-Mingorance A. Perez De Rozas A. (2020). *mcr*-colistin resistance genes mobilized by IncX4, IncHI2, and IncI2 plasmids in *Escherichia coli* of pigs and white stork in Spain. *Front. Microbiol.* 10:3072. 10.3389/fmicb.2019.03072 32010114 PMC6978640

[B27] Olaitan A. O. Morand S. Rolain J. M. (2014). Mechanisms of polymyxin resistance: acquired and intrinsic resistance in bacteria. *Front. Microbiol.* 5:643. 10.3389/fmicb.2014.00643 25505462 PMC4244539

[B28] Oteo J. Diestra K. Juan C. Bautista V. Novais A. Perez-Vazquez M. (2009). Extended-spectrum beta-lactamase-producing *Escherichia coli* in Spain belong to a large variety of multilocus sequence typing types, including ST10 complex/A, ST23 complex/A and ST131/B2. *Int. J. Antimicrob. Agents* 34 173–176. 10.1016/j.ijantimicag.2009.03.006 19464856

[B29] Papa-Ezdra R. Grill Diaz F. Vieytes M. García-Fulgueiras V. Caiata L. Ávila P. (2020). First three *Escherichia coli* isolates harbouring *mcr-1* in Uruguay. *J. Glob. Antimicrob. Resist.* 20 187–190. 10.1016/j.jgar.2019.07.016 31336172

[B30] Patiño-Navarrete R. Rosinski-Chupin I. Cabanel N. Gauthier L. Takissian J. Madec J. (2020). Stepwise evolution and convergent recombination underlie the global dissemination of carbapenemase-producing *Escherichia coli*. *Genome Med.* 12:10. 10.1186/s13073-019-0699-6 31955713 PMC6970295

[B31] Poirel L. Jayol A. Nordmann P. (2017). Polymyxins: antibacterial activity, susceptibility testing, and resistance mechanisms encoded by plasmids or chromosomes. *Clin. Microbiol. Rev.* 30 557–596. 10.1128/CMR.00064-16 28275006 PMC5355641

[B32] Poirel L. Kieffer N. Brink A. Coetze J. Jayol A. Nordmann P. (2016). Genetic features of MCR-1-producing colistin-resistant *Escherichia coli* isolates in South Africa. *Antimicrob Agents Chemother.* 60 4394–4397. 10.1128/AAC.00444-16 27161623 PMC4914673

[B33] Porse A. Schønning K. Munck C. Sommer M. O. A. (2016). Survival and evolution of a large multidrug resistance plasmid in new clinical bacterial hosts. *Mol. Biol. Evol.* 33 2860–2873. 10.1093/molbev/msw163 27501945 PMC5062321

[B34] Rossi F. Girardello R. Morais C. Cury A. P. Martins L. F. Silva A. M. (2017). Plasmid-mediated *mcr-1* in carbapenem-susceptible *Escherichia coli* ST156 causing a blood infection: an unnoticeable spread of colistin resistance in Brazil? *Clinics* 72 642–644. 10.6061/clinics/2017(10)09 29160428 PMC5666441

[B35] Sellera F. P. Fernandes M. R. Sartori L. Carvalho M. P. Esposito F. Nascimento C. L. (2017). *Escherichia coli* carrying IncX4 plasmid-mediated *mcr-1* and *bla*CTX-M genes in infected migratory Magellanic penguins (*Spheniscus magellanicus*). *J. Antimicrob. Chemother.* 72 1255–1256. 10.1093/jac/dkw543 28031274

[B36] Shen C. Feng S. Chen H. Dai M. Paterson D. L. Zheng X. (2018). Transmission of *mcr-1*-producing multidrug-resistant *Enterobacteriaceae* in public transportation in Guangzhou, China. *Clin. Infect. Dis.* 67 S217–S224. 10.1093/cid/ciy661 30423047

[B37] Snesrud E. He S. Chandler M. Dekker J. P. Hickman A. B. Mcgann P. (2016). A model for transposition of the colistin resistance gene *mcr-1* by IS*Apl1*. *Antimicrob. Agents Chemother.* 60 1416–1457. 10.1128/AAC.01457-16 27620479 PMC5075121

[B38] Sullivan M. J. Petty N. K. Beatson S. A. (2011). Easyfig: a genome comparison visualizer. *Bioinformatics* 27 1009–1010. 10.1093/bioinformatics/btr039 21278367 PMC3065679

[B39] Sun J. Yang R. S. Zhang Q. Feng Y. Fang L. X. (2016). Co-transfer of *bla*NDM-5 and *mcr-1* by an IncX3-X4 hybrid plasmid in *Escherichia coli*. *Nat. Microbiol.* 1:16176. 10.1038/nmicrobiol.2016.176 27668643

[B40] Wang Q. Sun J. Li J. Ding Y. Li X. Lin J. (2017). Expanding landscapes of the diversified *mcr-1*-bearing plasmid reservoirs. *Microbiome* 5:70. 10.1186/s40168-017-0288-0 28683827 PMC5500976

[B41] Wang R. van Dorp L. Shaw L. P. Bradley P. Wang Q. Wang X. (2018). The global distribution and spread of the mobilized colistin resistance gene *mcr-1*. *Nat. Commun.* 9:1179. 10.1038/s41467-018-03205-z 29563494 PMC5862964

[B42] Wick R. R. Judd L. M. Gorrie C. L. Holt K. E. (2017). Unicycler: resolving bacterial genome assemblies from short and long sequencing reads. *PloS. Comput. Biol.* 13:e1005595. 10.1371/journal.pcbi.1005595 28594827 PMC5481147

[B43] Xavier B. B. Lammens C. Butaye P. Goossens H. Malhotra-Kumar S. (2016). Complete sequence of an IncFII plasmid harbouring the colistin resistance gene *mcr-1* isolated from Belgian pig farms. *J. Antimicrob. Chemother.* 71 2342–2344. 10.1093/jac/dkw191 27261261

[B44] Yang R. Feng Y. Lv X. Duan J. Chen J. Fang L. (2016). Emergence of NDM-5- and MCR-1-producing *Escherichia coli* clones ST648 and ST156 from a single muscovy duck (*Cairina moschata*). *Antimicrob. Agents Chemother.* 60 6899–6902. 10.1128/AAC.01365-16 27550364 PMC5075103

[B45] Zhang C. Feng Y. Liu F. Jiang H. Qu Z. Lei M. (2017). A phage-like IncY plasmid carrying the *mcr-1* gene in *Escherichia coli* from a pig farm in China. *Antimicrob. Agents Chemother.* 61 e02035–16. 10.1128/AAC.02035-16 28031198 PMC5328573

[B46] Zhi C. Lv L. Yu L. Doi Y. Liu J. (2016). Dissemination of the *mcr-1* colistin resistance gene. *Lancet Infect. Dis.* 16 292–293. 10.1016/S1473-3099(16)00063-3 26973307

[B47] Zhong L. Phan H. T. T. Shen C. Vihta K. Sheppard A. E. Huang X. (2018). High rates of human fecal carriage of *mcr-1*–positive multidrug-resistant *Enterobacteriaceae* emerge in China in association with successful plasmid families. *Clin. Infect. Dis.* 66 676–685. 10.1093/cid/cix885 29040419 PMC5848316

[B48] Zhou Z. Alikhan N. Sergeant M. J. Luhmann N. Vaz C. Francisco A. P. (2018). GrapeTree: visualization of core genomic relationships among 100,000 bacterial pathogens. *Genome Res.* 28 1395–1404. 10.1101/gr.232397.117 30049790 PMC6120633

[B49] Zurfluh K. Klumpp J. Nüesch-Inderbinen M. Stephan R. (2016). Full-Length nucleotide sequences of *mcr-1*-harboring plasmids isolated from extended-spectrum-β-lactamase-producing *Escherichia coli* isolates of different origins. *Antimicrob. Agents Chemother.* 60 5589–5591. 10.1128/AAC.00935-16 27324774 PMC4997865

