# Supplementary material for: Genetic Features of Plasmid- and Chromosome-Mediated mcr-1 in Escherichia coli Isolates From Animal Organs With Lesions
Source: Front Microbiol. 2021 Aug 5;12:707332. doi: 10.3389/fmicb.2021.707332 (PMC8386294; doi:10.3389/fmicb.2021.707332)
Supplement: Supplementary file 1 [file Data_Sheet_1.docx]

Supplementary Material

**Table S1.** The MICs of 24 MPECs against 14 antimicrobials

| Strain No. | Year | MIC (μg/mL) | | | | | | | | | | | | | |
| --- | --- | --- | --- | --- | --- | --- | --- | --- | --- | --- | --- | --- | --- | --- | --- |
|  |  | AMP | CAZ | CTX | ATM | TET | DOX | CIP | ENR | GEN | SMZ | FOS | FFC | COL | MEM |
| 19-5 | 2019 | 512^R^ | >512 ^R^ | 16 ^R^ | 16 ^R^ | 512 ^R^ | 32 ^R^ | 64 ^R^ | 128 ^R^ | 256 ^R^ | >512 ^R^ | 128 ^I^ | >512 ^R^ | 4 ^R^ | < 0.25^S^ |
| 19-1 | 2019 | 128 ^R^ | 256 ^R^ | 0.5 ^S^ | 0.5 ^S^ | 128 ^R^ | 16 ^R^ | 64 ^R^ | 64 ^R^ | 16 ^R^ | >512 ^R^ | 4 ^S^ | 512 ^R^ | 2 ^R^ | < 0.25 ^S^ |
| B-1 | 2018 | >512 ^R^ | >512 ^R^ | >512 ^R^ | 512 ^R^ | 256 ^R^ | 32 ^R^ | 128 ^R^ | 128 ^R^ | 128 ^R^ | >512 ^R^ | >512 ^R^ | 16 ^R^ | 2 ^R^ | < 0.25 ^S^ |
| B-2 | 2018 | >512 ^R^ | >512 ^R^ | >512 ^R^ | 512 ^R^ | 256 ^R^ | 32 ^R^ | 256 ^R^ | 128 ^R^ | 128 ^R^ | >512 ^R^ | >512 ^R^ | 16 ^R^ | 4 ^R^ | < 0.25 ^S^ |
| B-3 | 2018 | >512 ^R^ | >512 ^R^ | 512 ^R^ | 64 ^R^ | 256 ^R^ | 16 ^R^ | 128 ^R^ | 64 ^R^ | 64 ^R^ | >512 ^R^ | >512 ^R^ | 8 ^R^ | 2 ^R^ | < 0.25 ^S^ |
| B-9 | 2018 | >512 ^R^ | >512 ^R^ | 512 ^R^ | 64 ^R^ | 256 ^R^ | 16 ^R^ | 128 ^R^ | 128 ^R^ | 256 ^R^ | >512 ^R^ | >512 ^R^ | 16 ^R^ | 2 ^R^ | < 0.25 ^S^ |
| 18-0 | 2018 | 2 ^S^ | 32 ^R^ | < 0.25 ^S^ | < 0.25 ^S^ | 1 ^S^ | 2 ^S^ | 64 ^R^ | 64 ^R^ | 1 ^S^ | >512 ^R^ | 32 ^S^ | 16 ^R^ | 2 ^R^ | < 0.25 ^S^ |
| 18-10 | 2018 | >512 ^R^ | >512 ^R^ | >512 ^R^ | 256 ^R^ | 256 ^R^ | 16 ^R^ | 128 ^R^ | 256 ^R^ | 1 ^S^ | >512 ^R^ | >512 ^R^ | >512 ^R^ | 2 ^R^ | < 0.25 ^S^ |
| 18-14 | 2018 | >512 ^R^ | >512 ^R^ | >512 ^R^ | 512 ^R^ | 256 ^R^ | 16 ^R^ | 128 ^R^ | 64 ^R^ | 128 ^R^ | >512 ^R^ | >512 ^R^ | 2 ^S^ | 2 ^R^ | < 0.25 ^S^ |
| 18-16 | 2018 | >512 ^R^ | >512 ^R^ | >512 ^R^ | 512 ^R^ | 512 ^R^ | 16 ^R^ | 64 ^R^ | 64 ^R^ | 64 ^R^ | >512 ^R^ | >512 ^R^ | 16 ^R^ | 2 ^R^ | < 0.25 ^S^ |
| S-4 | 2017 | >512 ^R^ | 512 ^R^ | >512 ^R^ | 128 ^R^ | 512 ^R^ | 64 ^R^ | 256 ^R^ | 256 ^R^ | 128 ^R^ | >512 ^R^ | 64 ^S^ | >512 ^R^ | 4 ^R^ | < 0.25 ^S^ |
| S-11 | 2017 | >512 ^R^ | >512 ^R^ | 512 ^R^ | 128 ^R^ | 32 ^R^ | 8 ^I^ | 4 ^R^ | 4 ^R^ | 128 ^R^ | >512 ^R^ | >512 ^R^ | 512 ^R^ | 2 ^R^ | < 0.25 ^S^ |
| I-R | 2017 | 512 ^R^ | 512 ^R^ | >512 ^R^ | >512 ^R^ | 64 ^R^ | 16 ^R^ | 2 ^R^ | 4 ^R^ | 2 ^S^ | >512 ^R^ | 64 ^S^ | 256 ^R^ | 4 ^R^ | < 0.25 ^S^ |
| A-3 | 2016 | >512 ^R^ | 16 ^R^ | >512 ^R^ | 512 ^R^ | 256 ^R^ | 64 ^R^ | 256 ^R^ | 4 ^R^ | 2 ^S^ | >512 ^R^ | >512 ^R^ | >512 ^R^ | 4 ^R^ | < 0.25 ^S^ |
| J-1 | 2016 | >512 ^R^ | >512 ^R^ | >512 ^R^ | 128 ^R^ | 128 ^R^ | 16 ^R^ | 128 ^R^ | 256 ^R^ | 128 ^R^ | >512 ^R^ | >512 ^R^ | 512 ^R^ | 4 ^R^ | 64 ^R^ |
| J-2 | 2016 | >512 ^R^ | >512 ^R^ | >512 ^R^ | 256 ^R^ | 128 ^R^ | 16 ^R^ | 128 ^R^ | 256 ^R^ | 128 ^R^ | >512 ^R^ | >512 ^R^ | 512 ^R^ | 4 ^R^ | 32 ^R^ |
| J-3 | 2016 | >512 ^R^ | >512 ^R^ | >512 ^R^ | 128 ^R^ | 128 ^R^ | 32 ^R^ | 256 ^R^ | 256 ^R^ | 128 ^R^ | >512 ^R^ | >512 ^R^ | 512 ^R^ | 4 ^R^ | 64 ^R^ |
| J-4 | 2016 | >512 ^R^ | >512 ^R^ | >512 ^R^ | 256 ^R^ | 128 ^R^ | 16 ^R^ | 128 ^R^ | 256 ^R^ | 128 ^R^ | >512 ^R^ | >512 ^R^ | 512 ^R^ | 4 ^R^ | 128 ^R^ |
| J-5 | 2016 | >512 ^R^ | >512 ^R^ | >512 ^R^ | 512 ^R^ | 256 ^R^ | 16 ^R^ | 128 ^R^ | 256 ^R^ | 128 ^R^ | >512 ^R^ | >512 ^R^ | 512 ^R^ | 4 ^R^ | 128 ^R^ |
| J-6 | 2016 | >512 ^R^ | >512 ^R^ | >512 ^R^ | 256 ^R^ | 128 ^R^ | 16 ^R^ | 128 ^R^ | 256 ^R^ | 256 ^R^ | >512 ^R^ | >512 ^R^ | 512 ^R^ | 4 ^R^ | 64 ^R^ |
| J-7 | 2016 | >512 ^R^ | >512 ^R^ | >512 ^R^ | 256 ^R^ | 128 ^R^ | 16 ^R^ | 128 ^R^ | 256 ^R^ | 256 ^R^ | >512 ^R^ | >512 ^R^ | >512 ^R^ | 4 ^R^ | 32 ^R^ |
| J-8 | 2016 | >512 ^R^ | >512 ^R^ | >512 ^R^ | 256 ^R^ | 128 ^R^ | 16 ^R^ | 128 ^R^ | 256 ^R^ | 256 ^R^ | >512 ^R^ | >512 ^R^ | 512 ^R^ | 4 ^R^ | 64 ^R^ |
| J-9 | 2016 | >512 ^R^ | >512 ^R^ | >512 ^R^ | >512 ^R^ | 256 ^R^ | 32 ^R^ | 32 ^R^ | 256 ^R^ | 8 ^I^ | >512 ^R^ | >512 ^R^ | >512 ^R^ | 4 ^R^ | < 0.25 ^S^ |
| N-14 | 2016 | >512 ^R^ | >512 ^R^ | >512 ^R^ | 256 ^R^ | 128 ^R^ | 16 ^R^ | 128 ^R^ | 256 ^R^ | 256 ^R^ | >512 ^R^ | >512 ^R^ | >512 ^R^ | 4 ^R^ | 64 ^R^ |

Ampicillin (AMP), ceftazidime (CAZ), cefotaxime (CTX), aztreonam (AZM), tetracycline (TET), doxycycline (DOX), ciprofloxacin (CIP), enrofloxacin (ENR), gentamicin (GEN), sulfamethoxazole (SMZ), fosfomycin (FOS), florfenicol (FFC), colistin (COL), meropenem (MEM). S, susceptible; I, intermediate; R, resistant.

**Table S2.** Origin and genetic diversity of 24 MPECs isolated from animal organs with lesions

| **Strain** | **Origin** | **Source (Organ)** | **ST** | **ST (CC)** |
| --- | --- | --- | --- | --- |
| J-1 | Poultry | Intestinal content (Intestine) | 156 | 156 |
| J-2 | Poultry | Intestinal content (Intestine) | 156 |  |
| J-3 | Poultry | Intestinal content (Intestine) | 156 |  |
| J-4 | Poultry | Intestinal content (Intestine) | 156 |  |
| J-5 | Poultry | Intestinal content (Intestine) | 156 |  |
| J-6 | Poultry | Intestinal content (Intestine) | 156 |  |
| J-7 | Poultry | Intestinal content (Intestine) | 156 |  |
| J-8 | Poultry | Intestinal content (Intestine) | 156 |  |
| N-14 | Bovine | uterine rinsing fluid (Uterus) | 156 |  |
| 18-10 | Swine | Intestinal content (Intestine) | 156 |  |
| 18-14 | Swine | Intestinal content (Intestine) | 448 | 448 |
| 18-16 | Swine | Intestinal content (Intestine) | 448 |  |
| B-1 | Swine | Intestinal content (Intestine) | 448 |  |
| B-2 | Swine | Intestinal content (Intestine) | 448 |  |
| B-3 | Swine | Intestinal content (Intestine) | 448 |  |
| B-9 | Swine | Intestinal content (Intestine) | 448 |  |
| S-4 | Swine | Intestinal content (Intestine) | 10 | 10 |
| 18-0 | Swine | Intestinal content (Intestine) | 48 |  |
| 19-5 | Swine | Pericardial Fluid (Heart) | 48 |  |
| 19-1 | Poultry | hepatic tissue (Liver) | 5454 | 206 |
| J-9 | Poultry | hepatic tissue (Liver) | 602 | 446 |
| A-3 | Swine | hepatic tissue (Liver) | 1403 | 278 |
| S-11 | Swine | hepatic tissue (Liver) | 10596 | ‒ |
| I-R | Swine | hepatic tissue (Liver) | 2179 | ‒ |

“‒”, not detected.
